# A Phenomenological Study of Educators’ Experience After a Year of the COVID-19 Pandemic

**DOI:** 10.3389/fpsyg.2022.869687

**Published:** 2022-05-27

**Authors:** Nagaletchimee Annamalai, Radzuwan Ab Rashid, Hadeel Saed, Omar Ali Al-Smadi, Baderaddin Yassin

**Affiliations:** ^1^English Section, School of Distance Education, Universiti Sains Malaysia, George Town, Malaysia; ^2^Faculty of Languages and Communication, Universiti Sultan Zainal Abidin, Kuala Terengganu, Malaysia; ^3^Department of English Language and Translation, Applied Science Private University, Amman, Jordan; ^4^English Language Department, University of Ha’il, Ha’il, Saudi Arabia; ^5^Al-Ghad International Colleges for Applied Medical Sciences, Riyadh, Saudi Arabia

**Keywords:** TPACK, phenomenology, COVID-19 pandemic, ICT tool, online learning (OL), online teaching and learning

## Abstract

This phenomenological study investigated educators’ lived experiences of teaching online in higher institutions in Malaysia. Data, which was generated through semi-structured interviews with 20 lecturers from three universities in the country, was analysed based on the thematic analysis approach guided by the Technological, Pedagogical, and Content Knowledge (TPACK)-self-efficacy framework. The findings revealed that after a year of teaching online, the potential of technology has been acknowledged by the educators after some trials and constraints were addressed. The domains related to Technology Content Knowledge (TCK), Technology Pedagogical Knowledge (TPK), and TPACK were evident in the findings. However, Pedagogical Content Knowledge (PCK) was not given emphasis, hence this manuscript argues that educators need to be constantly reminded of the significance of PCK. The findings discussed in this manuscript can be a helpful guide for educators when there is a need for them to teach online.

## Introduction

The COVID-19 pandemic is a global phenomenon that has transformed human endeavour and inadvertently accelerated and extended online education in higher educational institutions (HEI). The abrupt shift from the traditional lecture-based classrooms to the virtual environments is the “largest unplanned educational experiment ever undertaken” (Day et al., 2021, p. 1). During this precarious time, educators had to: (i) transit to online instruction (online teaching and learning or OTL, henceforth) with little or no preparation, (ii) conduct online instruction under duress situations, and (iii) endure uncertainty about the online instruction whether it is a temporary or permanent measure (Cutri et al., 2020). For some educators, it was a matter of drawing on their online experiences, and for some, it was a new learning experience as they had very little exposure to online instruction (Had and Rashid, 2019; Azizan et al., 2020; Dew et al., 2021). Hodges et al. (2020) demonstrated that adequately planned online learning experiences are not the same as online lessons’ response during a crisis. These researchers further define online teaching practices during this pandemic as emergency remote teaching (ERT) since it contrasts with the quality of the planned online learning practices.

In general, although educators experience “forced readiness” (Cutri et al., 2020, p. 533), they were optimistic and willing to revise their teaching and learning ideas to cater to the use of technology during the pandemic. We assert that the construct of optimism might be productively considered as part of the affective domains of faculty online readiness. The COVID-19 pandemic has propelled the predicted trends of HEI future directions underscored by digital technology, active-based learning models, and lifelong learning.

Online teaching practices during the initial stage of the COVID-19 pandemic have documented several significant findings, including hurdles accounted for by educators with the Internet connection, technology equipment, time taken to develop their teaching activities, anxiety and institutional support (Giovannella and Passarelli, 2020; Tartavulea et al., 2020; Annamalai, 2021). These studies have developed an oasis of knowledge on the strengths and barriers of going online during a precarious or tragic situation. However, limited studies are available to identify the shift in teaching practices during the pandemic after a year of experience in online teaching without a traditional lecture-based classroom. We argue that attention must be given to how educators experienced online teaching after a year of the COVID-19 pandemic, i.e., Are they progressing well? If yes, how? If not, why? And what can be done in helping them to have a better experience? Understanding educators’ teaching practices would be a solid basis to understand educators’ adaptation of teaching patterns to teaching and learning, and their measures to respond to tragic situations. Such findings could clarify the research findings–amid the challenges, what experiences motivate the lecturers to continue teaching during the pandemic.

Furthermore, there are debates and much uncertainty as to whether online teaching and learning practices offered by HEIs will be implemented in the future and to what extent the virtual learning environment will be a sustainable initiative. In these circumstances, it becomes more critical to conduct a phenomenological study on educators’ lived experiences of teaching online after a year of the COVID-19 pandemic. The findings could assist educational authorities and institutions to understand the educators’ difficulties and potentially improve the online teaching practices. This manuscript focuses on educators in Malaysia, but the lessons learned can guide them beyond its borders, particularly in developing countries.

The current study employed hermeneutic phenomenology to explore a complex phenomenon worthy of a thorough and detailed investigation. Phenomenology is considered a unique approach with its own theoretical and philosophical approach that differs from other approaches. It is the systematic study of lived experience and its inner meanings. Hermeneutic phenomenology as a research methodology was employed to uncover the distinctiveness of individuals’ experiences during the COVID-19 pandemic and what those experiences meant to educators. To the best of our knowledge, there is no hermeneutic phenomenological study of educators’ experience after a year of the COVID-19 pandemic, particularly involving Malaysian educators. The situational aspects of the interviewees were pertinent to the investigation since the understanding of a phenomenon (i.e., educator’s online teaching and learning experiences) had to be connected with a specific context in which the phenomenon was experienced (Englander, 2012). Thus, this study yields meaningful findings and conclusions that contribute to the field and extends the literature on teacher educators’ readiness during the COVID-19 pandemic. This study seeks to address the following research question: What is it like for university educators to teach online after a year of the COVID-19 pandemic?

### Theoretical Framework: Technological, Pedagogical, and Content Knowledge

The abrupt transition to online teaching and learning (OTL) has led educators to use technologies and relevant tools available to them to ensure continuity in their teaching practices (Annamalai et al., 2021). In this study, the Technological, Pedagogical, and Content Knowledge (TPACK) developed by Koehler and Mishra (2009) was employed to understand the educators teaching practices after a year of the COVID-19 pandemic. The TPACK model is based on the Pedagogical, Content, and Knowledge (PCK) framework developed by Shulman (1987), emphasising content knowledge of a subject and pedagogical knowledge. PCK is fundamental for effective teaching practices (Nor and Ab Rashid, 2018). With the inclusion of technology, a four-domain model, underlined by three main concepts, was initiated by Mishra and Koehler (2006). The three concepts are (i) Technological Pedagogical Knowledge (TPK)-integrating technological tools with pedagogical practices; (ii) Technological Content Knowledge (TCK)–integrating technology to specific content; (iii) PCK–integration of pedagogy and content knowledge, and (iv) Technological Pedagogical Content Knowledge (TPACK) technology integrated with pedagogical approaches and concept representations. According to Mishra and Koehler (2006), these domains are context-bound, and no single technology can be put in all the instructional situations. As educators employ specific tools for their teaching and learning activities, they are enhancing the TPACK model. Mishra and Koehler (2006) vehemently argued that the separate reference to the constructs of TPACK does not serve the purpose of effective teaching. Each of the constructs complements each other, and no constructs can stand on their own independently.

Technological, pedagogical, and content knowledge model is context-dependent with “unique internal and external contexts” (Porras-Hernandez and Salinas-Amescua, 2013, p. 231). Therefore, in the current study, TPACK-self efficacy serves as a lens to understand educators’ experiences and perspectives after a year. Teacher self-efficacy is defined as “the teacher’s belief in his or her capability to organise and execute a course of action required to accomplish a specific teaching task in a particular context” (Tschannen-Moran et al., 1998, p. 233). In other words, educators should also have the confidence to integrate technology tools into their teaching. It emphasises on educators’ faith and beliefs in integrating technology rather than their knowledge or skills acquired in technology (Al-Awidi and Alghazo, 2012). An educator with a firmer self-efficacy belief about technology integration will probably use educational technology in their teaching and learning activities (Anderson et al., 2011). Tondeur et al. (2012) have highlighted that educators should integrate proper technology models, and knowledge should be presented to develop self–efficacy. In response to Tondeur et al.’s (2012) recommendation, we concur that in terms of TPACK and self-efficacy, there ought to be new directions and focus of research. Therefore, a nuanced perspective is required to understand the TPACK-self efficacy model during the COVID-19 pandemic. Although the TPACK model has been widely studied during the pre-COVID-19 pandemic, it remains an understudied area during the COVID-19 pandemic hence our motivation for undertaking the current study.

## Studies Related to Online Learning During the COVID-19 Pandemic

There is a vast difference between online teaching during the pandemic and the pre-COVID era. This is because, in the pre-COVID age, educators had sufficient time to prepare their teaching approaches, materials, and assessment. However, little time and minimum resources are available for them to teach in the virtual environment during the pandemic. Therefore, educators who lack online teaching experience are more likely to deliver their online teaching using teacher-centred approaches rather than student-centred approaches. Regarding educators’ expertise during the initial stage of online learning, Giovannella and Passarelli (2020) conducted a survey with 546 educators in a university in Italy. They reported that university educators are more negative and more pessimistic about adapting online teaching than high school teachers. Tartavulea et al. (2020) surveyed educators from 13 European countries and discovered that educators are quick and positioned in adapting to online teaching. Hjelsvold et al. (2020) conducted interviews with 22 educators in Norway who were optimistic about teaching online but reported that they lack pedagogical competence. In Turkey, Eroglu and Senol (2021) discovered that ERT was conducted using various online applications such as Zoom, YouTube, and WhatsApp. The study reported that ERT was ineffective because of the low student motivation, insufficient infrastructure, inappropriate planning and low socioeconomic status. Aladsani (2021) qualitative survey reported educators’ feelings and challenges during the transition to an online learning environment. The study reported on the effect of the local culture and the millennial generation student status on students’ engagement.

Several studies have also utilised the TPACK framework during the initial stage of the COVID-19 pandemic. For example, Rap et al. (2020) conducted a survey to identify Chemistry teachers’ difficulties and strategies for OTL. Mohamad Nasri et al. (2020) used reflection and examined teaching experience based on the TPACK framework and suggested that students and educators need to acquire the knowledge of TPACK to ensure effective learning. A survey conducted by Rabaglietti et al. (2021) examined European teachers’ self-efficacy and reported decreased self-efficacy. The authors found that self-efficacy appeared to mediate educators’ stress and difficulties in distance learning. Another study on self-efficacy by Dolighan and Owen (2021) reported a high level of self-efficacy after the school teachers have attended additional courses for online teaching. Although these studies were related to self-efficacy, the studies were focused on high school teachers.

Generally, most of the studies reviewed above have examined studies during the initial transition to online learning. While these studies have yielded significant findings in prevalence rates, readiness, patterns of behaviours, coping strategies and causes, there is a need for more studies to provide a rich and in-depth investigation of the lived experiences of the educators. Damşa et al. (2021) indicated that studies related to educators transitioning to online learning during the COVID-19 pandemic are limited. Most studies at the initial stage of online learning were focused on the quality and emergency of online learning (Hussein et al., 2020). More informed and effective online teaching is pertinent to understanding its potential.

The current study differs from previous studies related to the COVID-19 pandemic which are not designed to examine teachers’ experiences after a year. We believe that the quality of online teaching would have developed in a year, and there might be significant contributions after trial and error within a year. This gap in the literature has not gone unnoticed. Mohamad Nasri et al. (2020) have called for more in-depth qualitative studies on the TPACK framework and educators to identify why and how educators engage in specific actions and behaviours. Thus, more investigations are needed on this aspect. In recognising this, we were keen to investigate the educators’ experiences of OTL after a year of pandemic.

## Methodology

This study employed a hermeneutic phenomenological research design. This design is mainly used to enhance the interpretive element to elucidate assumptions and meanings in the transcribed texts of participants’ interviews where they themselves may have difficulty expressing, hence offering a rich detailed and dense description of the phenomenon under investigation (Van Manen, 2017).

### Participants

Using a purposive sampling approach, 20 participants were selected from three universities in Malaysia. They were the educators who experienced the phenomenon (teaching online from the beginning of the pandemic till a year). Ten of them were from the social science field, and the other 10 were from the applied science field. The fact that the participants were from different backround enabled us to capture the phenomenon from a “broader view” and gain “a community perspective” instead of “a case-by-case approach” (Bush and Amechi, 2019, p. 7). Participants’ names and identities were not revealed and given pseudonyms, as informed to them prior to the interview, to protect their privacy and encourage them to express themselves confidently and openly. The participants were given pseudonyms as PP1, PP2, PP3, and so forth. Before the data was collected, the participants were briefed on the nature of the study. They were informed that they would be able to withdraw at any juncture or phase of the study without any explanation.

### Data Collection

In the absence of the direct interviews due to social distancing and lockdown in Malaysia at the research time, we had a Webex (video conference) meeting. Each discussion *via* video conferencing was conducted individually for 30–45 min. Further, telephone calls were made to enhance the accuracy and adequacy of the data and for the member check purpose. Probes were frequently used to clarify their meaning and encourage in-depth explanations (Penner and McClement, 2008). The interview questions include: How do you feel about teaching online after a year of the COVID-19 pandemic? Can you explain to me about technology, pedagogy and content aspects of your online teaching so far?

We demonstrated the findings and their interpretations based on the TPACK model. We then presented the overall findings of the analysis and invited the participants to comment on our interpretation in our attempt to engage in “member checking” and increase the trustworthiness of this study. All participants agreed and acknowledged that the findings and interpretation were consistent, accurate and acceptable.

Hermeneutic phenomenology is not confined to single techniques of analysis. It is an interpretative method that includes several analytical activities (Bynum and Varpio, 2018). Following Bynum and Varpio’s (2018) suggestions, the researcher used Nvivo to analyse the data further. This software helped in organising the data thus facilitated data interpretation process. Analytical memos were used to record researchers’ thoughts during the data analysis. Analytical memos refer to the researchers’ ideas while analysing the data, and code memos as proofs of the thoughts (Saldaña, 2016). We maintained a clear orientation of the phenomenon under investigation during the analysis. The final step was, as in accordance with the procedures of the hermeneutic study, to consciously attempt to understand how the data gave meaning to the evolving understanding of the phenomenon under investigation. We then utilised the six steps of thematic analysis by Braun and Clarke (2006) in the categorisation of emerging themes in this study–(i) familiarisation of the data, (ii) generating initial codes, (iii) searching for themes, (iv) reviewing themes, (v) defining and naming themes and, (vi) producing the report.

### Trustworthiness of the Study

Member checking, referential adequacy, and peer briefing were established to ensure credibility. Member checking was conducted to establish truth in the findings by asking participants to read and comment on the interview transcriptions and their interpretations. If there were any misinterpretations in the data pointed out by participants, the idea or sentences were eliminated. Dependability explained to what extent research can be repeated in a similar setting (Erlandson et al., 1993). An audit trail was employed to achieve reliability. By describing the research procedures from the beginning of the study and reporting the findings, the audit trail was fulfilled. Transferability captures the extent to which the results of the study can be applied in other contexts. Detailed and thorough descriptions of the sampling and reporting of the findings were provided, which allowed judgement by the readers about transferability. All the processes were recorded so that the facts and assertions could be tracked to their sources. Investigator triangulation was achieved when three experienced lecturers in qualitative research coded the emerging themes and reached 90% agreement between the coders. Hence, the findings are reliable, convincing, and accurately reflect the actual situation.

## Data Analysis

In the following section, the themes that emerged are discussed. The interview excerpts are presented as original without amendments of grammar and sentence structure which is a common practice in a phenomenology to reflect the characteristics of natural data.

The themes were categorised based on the following:

i. Technological pedagogical knowledge (TPK), i.e., the means of incorporating technology with pedagogical knowledge. The emerging themes were related to problem-based learning, gamification, and flipped classroom.
ii. Technological content knowledge (TCK), i.e., integrating technology to particular content. The emerging themes were related to cloud base storing, sharing of course content, byte size learning.
iii. Pedagogical Content Knowledge (PCK), i.e., describing the integration between pedagogical practices and specific learning objectives.
iv. Technological pedagogical and content knowledge (TPACK), i.e., representing a strategic fit between technology and pedagogy for effective teaching. The emerging themes were related to self-determination, asynchronous, synchronous discussion, and minimal technical issues.

### Technological, Pedagogical, Content, and Knowledge

Most of the educators indicate that technological tools are used without much planning during the initial phase of Movement Control Order (MCO). However, after some time, changes were made accordingly. They realised that they lack knowledge on the appropriate tools for OTL as the transition was sudden. However, as participants begin to experience numerous OTLs during the last year, they have *“explored various technological tools and environments”* (PP7, PP11, PP9), and have familiarised themselves with new online technologies (PP3, PP4, PP5, PP13) to feel “*more confident”* (PP4, PP5) in using technology tools for their OTL practices. Basically, participants “*discovered many new technological tools that they have never encountered prior to the pandemic*” (PP18).

The limited exposure to technology tools at the beginning of their online teaching instils fear, uncertainty and burdens the educators. Self-determination is one of the factors that kept these educators exploring various tools despite the challenges experienced during the COVID-19 pandemic.

PP5 clarifies that:

*In the initial stage I used the heavy Webex software, and later change to Zoom which is more compatible with online teaching. Zoom software is easier to handle and functions smoothly using any version of laptop or PC.*

In one year of OTL, as a result of various exploration and continuous experiences of a total OTL, participants learned about and experimented with various new technologies that would be meaningful for different pedagogical purposes. They were able to explore different types of technologies to make teaching more interactive with asynchronous and synchronous discussion tools. Most of the educators mentioned the use of Kahoot (PP2, PP7, PP8, PP10), Google classroom (PP2, PP7, PP8, PP10), and YouTube (PP6, PP10) as useful tools for them. For synchronous discussion, they have used Webex, Zoom, and Teams (PP3, PP4, PP5). PP9 discovered that *“the use of Quiziz during lessons helps to improve interaction and socialisation in the classroom.”* PP8 preferred to use *“Kahoot, Quizlet, and Wooclap during the synchronous discussion and Google classroom for feedback especially for the written assignment.”*

Some of the participants reported that they were very committed and concerned about the effectiveness of their OTL. They invested: (i) money in technological tools to improve and enhance their capabilities to plan and conduct a more sophisticated OTL and, (ii) more time and effort on learning new knowledge and ideas related to technology and pedagogy. These are necessary for the participants to ensure their OTL is interactive, engaging and interesting, making their teaching more interactive, as explicated by these participants:

…*it is very difficult to explain notes with some simple sketches or writing during initial stage MCO because of the limited knowledge on technology-assisted language learning tools. I mostly rely on the software and applications which are compatible to my personal computers and easy on any bandwidth. It took time and I slowly invested in tools that made teaching much easier. Later I bought a digital tablet for writing and sketching during my lecture (PP5).*

*I have limited knowledge of technology-assisted language learning tools. However, after a year of experiencing online teaching, I am more aware of the use of technology tools to deliver content. I need to invest in some good quality webcam and microphone in my synchronous session. I explored the use of free online broadcasting tools too and tried to keep my presentation versatile according to the content (PP12).*

*I do not find any difficulties compared to the initial lockdown where I need to learn and explore many new things that I was not familiar with such as conducting online classes, preparing materials, recordings, assessments, etc., on my own. However, there are still many other things that I need to learn and improve (PP19).*

Their interest and commitment made them to *be “familiar with digital tools,”* (PP5) *“technical issues”* (PP7, PP9, PP10) and able to conduct the online classes with *“minimal technical issues”* (PP13, PP14). One participant concluded that *“I am more confident in using various technology tools now compared to last year (2020) but I need to explore the features more to make my lessons interesting and engaging”* (PP17).

### Technology Content Knowledge

Most of the participants explained that teaching online was compulsory at their university and they did not find this easy. However, it encouraged self-reliance and self-discovery of innovative tools for delivering the content. Educators discovered ways that were convenient and effective to convey their ideas. PP7 expressed that she “*believes in learning by doing”* and confronted that she *“got frustrated when teaching the students skills related to listening, speaking, and writing because they were not able to show [her] how they attempt the activities.”* A greater sense of achievement was evident as time went on. The teacher discovered tools that can facilitate interaction and discussions. She explained:

*After a year now, I have explored other pedagogical skills by utilising technological tools to assist me in teaching. It is a struggle but I select tools which are not too time-consuming. For example, Jamboard are good for speaking and writing classes. Whereas for interactive reading, I get students to highlight texts using annotation functions.*

The fear of uncertainty in delivering the content was expressed by most of the participants at the initial stage of MCO. As they adapt to virtual teaching they seem to be positive and took every opportunity and remain driven to teach even though online teaching has many challenges. A positive attitude kept them moving and exposed them to the realities of teaching during the COVID-19 pandemic. PP16 detailed that:

*I am improving my time management as an instructor. I am learning to prioritise tasks pertaining to content delivery, feedback sharing, asynchronous activity, class preparation time, assessment preparation, and assessing students’ ability. After a year, I learnt that some content delivery can be recorded and stored in the cloud for other students as a reference. This allows me more time to focus on content improvement and keeping content up to date. Activities can be easily stored in the cloud and readily retrieved when needed saving time and other resources along the way.*

One positive value shared by the teachers is the sharing of course content. There was an effort made by lecturers to develop content to suit the synchronous discussion. PP2 said that *“all content must follow the course learning outcomes and must be uploaded to cloud for sharing and access.”*

They were also generous in sharing their recorded teaching on YouTube for others to access freely. Educators displayed transformative conduct, engaged in professional development and experienced positive teaching outcomes of such conduct.

After a year of online teaching educators PP19 realised that:

*content needs to be compact and direct to the point as we do not want to waste students’ time. Students’ do not like a long lecture, as it can make them lose focus in online learning.*

They need more time to understand the online lesson compared to face to face classes, so, it is important to have synchronous and asynchronous classes to give them ample time to learn on their own and completed the activities prepared for them.

PP19 found that:

*After a year of going online, there has been a lot of changes in the way I run my virtual classes. I realised that keeping lessons short and simple is appealing to students. They have a limited attention span during virtual lessons than they would in face-to-face interaction. However, one thing remained true of the nature of lessons conducted during the initial lockdown periods and after a year; my students are not responsive during lessons, and they prefer sending a private message through messaging applications. I had to rely on “pin drop silences” as the way of saying that the lesson was understood.*

### Technology Pedagogy Knowledge

The following excerpts indicate educators’ adaptive practices where they go beyond their regular practices to experiments with new approaches. They shift their teaching to be *“more problem oriented where students are given some task or mini-projects to complete, and evaluation is conducted based on their abilities, creativity, and problem-solving skills”* (PP17). Educators felt that PBL (problem-based learning) is worthwhile as it gave opportunities for them to practice students’ centred learning where students *“conduct an online presentation on relevant topics,” “create own mind map based on the learned chapter*,” and do “*video recording”* (PP10).

Educators also planned and carried out fun game-based activities and flipped classroom learning to ensure students would be able to actively participate during the online classes. For example, PP8 detailed the fun game-based activities using tools such as *“Kahoot, Quizlet, and Wooclap to engage them in the instruction and to ensure interaction during the class”* (PP8). PP9 stressed that *“lecturers need to record their lectures before the class,”* where she recorded videos *“for a 1-h lecture.”* Her teaching practices now tend to focus on the flipped classroom and bite-size learning approaches. PP9 described her teaching practices,

…*after a year, I change the method to the flipped classroom. I gave short videos for lecture (15–20 min) and in the class, I encourage active participation using apps like Padlet, Jamboard, etc. I also prepared micro-credential modules as part of a course, I am teaching. In each module, I include teaching videos, learning activities, and assessments.*

Educators were also aware of the importance of informal interaction as students are isolated and learning remotely. PP10 details that:

*The early-stage to online teaching is purely lecture-based where no social interaction occurs with students. Most of the students seldomly talk or answer or open the webcam during lecture. However, I slowly improved the learning environment by initiating simple conversations with students apart from lecture notes to catch their attention before starting my lecture. Sharing jokes or funny stories is very difficult at an early stage where students’ responses are very poor in an online medium. However, over time I made sure they prepared for the class with good internet connectivity to avoid any interruption during class.*

Table 1 summarises the themes that emerge in describing the educators’ experiences in the initial phase of the COVID-19 pandemic and 1 year after going through the pandemic.

**TABLE 1:** Educators’ experiences in the initial phase and a year of the COVID-19 pandemic.

Initial phase of the pandemic	After a year of the pandemic
• Using technological tools without much planning • Having fear of online teaching • Having limited exposure to useful tools for online teaching • Feeling frustrating in attempting for an effective online teaching	• Exploring and experimenting with various tools • Feeling more confident in using online tools • Investing money on technological tools • Investing more time and effort to master online pedagogy Building self-reliance • Appreciating the feeling of self-discovery • Integrating problem based learning

## Discussion

This study aimed to contribute a better understanding of educators’ teaching experiences after a year of the COVID-19 pandemic. It is evident that the participants in this study have embraced the transition more thoughtfully after authentic experimental ground during the initial online practices.

It is obvious that the sudden transition during the initial stage of online learning left educators with little choice and respond purposefully to seek a solution for the immediate virtual learning (Campbell, 2020). However, the findings revealed that the educators have tried out various possibilities to overcome the challenges and contingencies with optimism. They seem to be more confident after a year of experience in online teaching. They opted for alternative solutions and managed constraints independently. Educators in this study, as suggested by Pressley et al. (2018), seem to have higher self-efficacy and are open to new teaching approaches, and are more determined to face challenges as they adapt to the online environment. With higher self-efficacy, educators have more favourable outcomes in their teaching practices because they are comfortable to scaffold and build relationships with students (Hajovsky et al., 2020). The initial research on educators’ self-efficacy during the COVID-19 pandemic suggested that many educators experienced a decrease in self efficacy due to stress (Rabaglietti et al., 2021) and burnout (Pellerone, 2021). However, the findings of the present study have provided a glimpse of positive attitudes and higher self-efficacy toward online teaching. Readiness to teach online refers to the state of preparation to teach online (Martin et al., 2019). It is for this reason that Kebritchi et al. (2017) state that sufficient time is needed to design and innovate online teaching practices and become competent and self-confident instructors (Kebritchi et al., 2017). Teachers’ positive attitude, commitment, and initiative have resulted in transforming and engaging in “pedagogical problem-solving and discovery about online teaching” (Kreber and Kanuka, 2006, p. 122).

Relating to TCK, lecturers were exposed to the realities of teaching during the pandemic. The environmental conditioning and teaching facilities can inspire their content delivery despite all the challenges. Educators were self-motivated and self-indulgent in their teaching practices during the pandemic. They have the natural tendency to shift to cloud-based storing and byte size learning. Educators’ ICT competencies are pertinent for successful online practices. Their effective use of technology tools in their teaching practices and their willingness to use online communication forms the basis of effective online teaching activities (Mukoviz et al., 2018). This implies that educators might decrease in efficacy in the new challenging demands, nevertheless, their efficacy improved over time as they learn to adapt to a new situation and take advantage of the available sources that are practical and relevant in their teaching practices (Sokal et al., 2020). Capitalising on various tools and resources (i.e., Kahoot, Google classroom, Zoom, YouTube, WhatsApp) and recognising the importance of byte size learning, the educators moved forward to the virtual environment and embraced the needs of 21st-century learning skills. Basically, the educators in this study were resourceful and utilised whatever they had to deliver their teaching. Technology tools and practices are intertwined and influence transformative potential and have the potential to initiate new practices (Damsa and Jornet, 2016). It can also be deduced that the more time the educators spend on technology tools and web resources, the higher the level of awareness of the online tools for teaching and interaction opportunities, and according to Firat and Bozkurt (2020), teachers who spend more time online are likely to have a higher level of awareness of online communication channels and interaction opportunities.

The instructors worked independently and build their confidence to think innovatively to enhance their TPK. Online Flipped classroom methods are gaining momentum during the pandemic since it provides students a lot of time and space to work on the task given before virtual face-to-face meeting. Although there is no physical proximity during the crisis, the synchronous discussion seems to be a well-studied teaching pedagogy, providing learners with pre-recorded didactic video lectures to be watched at the learner’s leisure before an in-person conference. These methods are argued to be more effective than traditional teaching methods to improve student learning performance (Hew and Lo, 2018). It is through this type of learning that educators can engage the students in interactive environments. Constructivist learning theory suggested by Vygotsky and Michael (1978) emphasised that interaction and collaboration are critical in knowledge construction. Similarly, Adedoyin and Soykan (2020) emphasised the need for education higher institutions to adapt to “constructivist, learner-centred, cooperative pedagogy” (p. 2). We conclude that the educators have attempted to create opportunities and the space for students to communicate and find solutions for problems (problem-based learning), where the learners apply new knowledge to different situations. Educators’ commitment and initiative in the domain related to TPK have emerged as a “victor ludorum amidst this chaos,” which implies that enhancing and enriching the quality of online teaching is critical during this time of crisis (Dhawan, 2020, p. 7). The effort of educators to discover available virtual resources is termed as “assemble an epistemic space” (Markauskaite and Goodyear, 2017) in which goals and needs of educators are addressed by applying and manipulating available digital resources and others expertise. The focus on TPK allowed active learning and a student-centred approach to take place. Education institutions tend to provide training in the utilisation of tools but during the pandemic, no instructors were taught on pedagogical practices or content delivery based on their disciplines (Estes, 2016). This is evident in this study as no participants mentioned training or workshops for pedagogical strategies and skills to promote students’ engagement in the virtual environment.

Technological, pedagogical, and content knowledge is also evident when asynchronous and synchronous tools were used in this study for effective teaching practices. According to Walker and Koralesky (2021), synchronous tools are more enjoyable and engaging and for participants who need encouragement. On the other hand, asynchronous tools provide more flexibility (Hodges et al., 2020). They talked about their confidence and elevated self-efficacy. Students’ peer support was evident and it acts as a change agent (Rogers, 2002) to increase professional development and to facilitate the transition to online learning.

Educators who spend more time in the online environment tend to discover online communication and interaction opportunities more effectively. Teachers who spend more time online are likely to have a higher level of awareness of online communication channels and interaction opportunities (Çınar et al., 2021). It is for this reason that Martin et al. (2019) assert that teaching online influences course design and facilitation. They need to be creative and willing to embrace changes in teaching and learning in the new normal, while discovering new possibilities and capabilities of teaching and learning (Tan et al., 2020).

The findings underscore that though technological tools are essential for online teaching practices during the pandemic, it is the non-technological factors such as pedagogical practices and content delivery that make a significant difference to engage learners. According to Mishra et al. (2020, p. 8) digital transformation is pertinent as well as developing a “curriculum that reflects the perceptible change in the content knowledge and learning experience of students as well as enables them to think critically.” The commitment and the educators’ collective agentic activities resulted in professional learning and collaboration. Adaptive teaching is considered a decisive feature of high-quality instruction (König et al., 2020). Teachers’ self-efficacy was also significant for providing task differentiation. These findings correspond with research that emphasises the importance of teacher competence in successfully attaining relevant educational goals (Kaiser and König, 2019).

**FIGURE 1:**
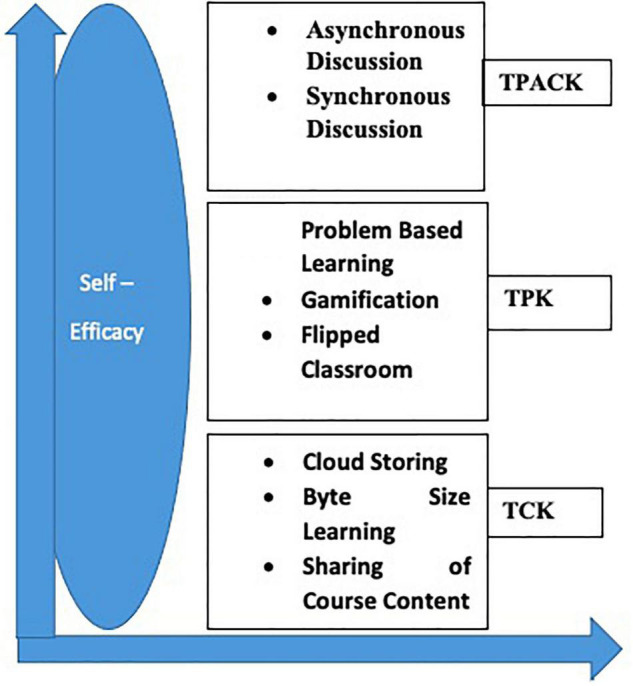
Online teaching experience after a year of the COVID-19.

Figure 1 illustrated the TPACK–self-efficacy component and the emerging themes in each domain.

What is still missing after a year of online teaching is PCK. PCK relates to teaching specific content. Educators in this study are more focused on getting their TCK, TPK, and TPACK. More effort is needed to focus on specific content (PCK). Since TCK and TPK have been established, more time and effort need to be prioritised to enhance PCK. Perhaps, educators’ teamwork on how to establish pedagogical practices in their subject matter is necessary. It is pertinent for educators to know the appropriate teaching approaches/pedagogical practices for their subject matter for effective learning to take place.

## Conclusion

This study contributes to our understanding that when educators are given more time to experiment with their teaching practices, they are more positive and ready to teach online. In other words, the more online learning experience teachers have, the readier they are for e-learning. Therefore, opportunities for an authentic experience with online teaching is beneficial in developing online teaching pedagogical skills. The findings point to endeavours in fostering educators’ competencies when it comes to online learning and not confined to general technology skills.

This study which is framed in a particular time (i.e., after a year of the pandemic) should encourage more studies involving different perspectives and contexts to further develop knowledge on how educators respond to changes caused by pandemic. Researching an ongoing pandemic is not static. By the time the present study makes it to publication, the realities of the world will change drastically. As such, it is crucial to remember, as Austin (2020) points out, “quality research of any kind takes time” (p. 9). Last but not least, we recognise the limitation of the present study, which other researchers may want to address in future studies. A large-scale survey or a mixed-method approach from different universities in Malaysia, which may reflect the scenario of overall OTL in the Malaysian universities should be considered by future researchers.

## Data Availability Statement

The raw data supporting the conclusions of this article will be made available by the authors, without undue reservation.

## Ethics Statement

Ethical review and approval was not required for the study on human participants in accordance with the local legislation and institutional requirements. The patients/participants provided their written informed consent to participate in this study.

## Author Contributions

NA designed the study and wrote the manuscript. RA, OA-S, BY, and HS helped in editing and adding more information to enhance the quality of the manuscript. All authors contributed to the article and approved the submitted version.

## Conflict of Interest

The authors declare that the research was conducted in the absence of any commercial or financial relationships that could be construed as a potential conflict of interest.

## Publisher’s Note

All claims expressed in this article are solely those of the authors and do not necessarily represent those of their affiliated organizations, or those of the publisher, the editors and the reviewers. Any product that may be evaluated in this article, or claim that may be made by its manufacturer, is not guaranteed or endorsed by the publisher.
